# cART prescription trends in a prospective HIV cohort in rural Tanzania from 2007 to 2011

**DOI:** 10.1186/1471-2334-14-90

**Published:** 2014-02-20

**Authors:** Fabian Christoph Franzeck, Emilio Letang, Geoffrey Mwaigomole, Boniphace Jullu, Tracy R Glass, Daniel Nyogea, Christoph Hatz, Marcel Tanner, Manuel Battegay

**Affiliations:** 1 Swiss Tropical and Public Health Institute (SwissTPH), Socinstrasse 57, CH-4051 Basel, Switzerland; 2 Ifakara Health Institute (IHI), Ifakara, United Republic of Tanzania; 3 Barcelona Centre for International Health Research (CRESIB-Hospital Clínic, Universitat de Barcelona), Barcelona, Spain; 4 University of Basel, Basel, Switzerland; 5 Division of Infectious Diseases and Hospital Epidemiology, University Hospital Basel, Basel, Switzerland

**Keywords:** cART, Regimen, Combination, Africa, Stavudine, HIV, AIDS

## Abstract

**Background:**

Since 2010, World Health Organization (WHO) guidelines discourage using stavudine in first-line regimens due to frequent and severe side effects. This study describes the implementation of this recommendation and trends in usage of antiretroviral therapy combinations in a cohort of HIV-positive patients in rural Tanzania.

**Methods:**

We analyzed longitudinal, prospectively collected clinical data of HIV-1 infected adults initiating antiretroviral therapy within the Kilombero Ulanga Antiretroviral Cohort (KIULARCO) in Ifakara, Tanzania from 2007-2011.

**Results:**

This analysis included data of 3008 patients. Median age was 38 (interquartile range [IQR] 31-45) years, 1962 (65.2%) of all subjects were female, and median CD4+ cell count at enrollment was 168 cells/mm^3^ (IQR 81-273). The percentage of prescriptions containing stavudine in initial regimens fell from a maximum of 75.3% in 2008 to 10.7% in 2011. TDF/FTC/EFV became available in 2009 and was used in 41.9% of patients initiating cART in 2011. An overall on-treatment analysis revealed that d4T/3TC/NVP and AZT/3TC/EFV were the most prescribed combinations in each year, including 2011 (674 [36.5%] and 641 [34.7%] patients, respectively). Of those receiving stavudine in 2011, 659 (89.1%) initiated it before 2011.

**Conclusions:**

Initial cART with stavudine declined to low levels according to recommendations but the overall use of stavudine remained substantial, as individuals already on cART containing stavudine were not changed to alternative drugs. Our findings highlight the critical need to exchange stavudine in treatment regimens of patients who initiated therapy in earlier years.

## Background

The revision of the World Health Organization (WHO) HIV treatment guidelines in 2010 brought several changes to the management of HIV patients [1]. Among them was a strong statement about progressing to less toxic antiretroviral drugs in first-line regimens, i.e. discouraging the use of stavudine in initial drug combinations. This drug was crucial in rolling out combined antiretroviral therapy (cART) in low-income setting as in Sub-Saharan Africa (SSA) for its low price and good efficacy in suppressing replication of HIV. However, severe side effects associated with stavudine use, such as peripheral neuropathy, lactic acidosis and lipodystrophy have been shown to be frequent and accumulating over time in several African cohorts [2,3]. In the United States, stavudine was already removed in 2004 from the list of preferred first-line antiretroviral drugs recommended by the US Department of Health and Human Services (DHHS) [4].

The goal of phasing out stavudine still competes in budgetary terms with other priorities in advancing cART as direct drug costs of stavudine were still at around 50% compared to alternative regimens in 2011 [5]. Recently, prices for generic formulations of more modern pharmaceuticals such as tenofovir have declined substantially. This should assist efforts to achieve the WHO goal of phasing out the use of stavudine containing regimens. In some settings, replacing stavudine with tenofovir or zidovudine was judged cost-effective [6-8] and further price reductions for these pharmaceuticals are to be expected.

Many national HIV programs have consecutively adopted the recommendations of the WHO, including the National AIDS Control Program (NACP) of Tanzania: In February 2009, the preferred regimen was changed from d4T/3TC/NVP to AZT/3TC/EFV and TDF/FTC/EFV was introduced as an alternative option [9]. However, d4T/3TC/NVP was still supported as a valid first-line combination for patients with anemia initiating therapy. No recommendation was issued to replace stavudine in existing prescription if it was tolerated without occurrence of severe side effects.

In order to describe how recommendations issued by the WHO eventually permeate to treating clinicians in low-income countries, this study describes prescription trends of antiretroviral therapy combinations in a large cohort of HIV patients in rural Tanzania over a five-year period.

## Methods

We analysed data of HIV-1 infected adults (≥ 18 years of age) initiating cART within the Kilombero Ulanga Antiretroviral Cohort (KIULARCO) at the chronic disease clinic at the St. Francis Referral Hospital in Ifakara. The hospital is the most important health care facility in the rural Kilombero and Ulanga Districts of the Morogoro Region in Southern Tanzania, providing treatment and care for a population of about 600,000 inhabitants. Demographic, clinical and laboratory data were prospectively collected from consenting patients at each consultation at the clinic. Medical doctors and clinical officers filled in standardized paper forms which consecutively where double entered into an electronic database (DMSys, Sigmasoft, Illinois, USA). Treatment failure was defined clinically or immunologically according to WHO criteria (50% drop in CD4 count from peak value within 6 months, or return to pre-ART baseline CD4 count or lower). Antiretroviral therapy combinations used for cases of treatment failure are referred to as second-line treatment regimens. Data collected from the 1st of January 2007 until the 31st of December 2011 were included in the analysis. In case a subject received > 1 regimen in one calendar year, all combinations prescribed during that year were included in the overall prescription analysis. Patients who initiated cART before 2007 were included in the overall prescription analysis. There was no active tracking mechanism of patients lost to follow-up during the study period. Continuous variables are expressed as median (interquartile range) and frequencies (percentages) for categorical variables. For calculation of p-values for trend, a chi-square statistic for trend was used. All statistical analyses were performed using Stata 11.2 (StataCorp, Texas, USA).

### Ethical statement

All subjects included were given oral and written information about the collection of their clinical data and signed an informed consent form. Ethical approval was granted by the local Ethical Committee of the Ifakara Health Institute and the Medical Research Coordination Committee of the National Institute of Medical Research of Tanzania.

## Results

### Patients characteristics

**Figure 1:**
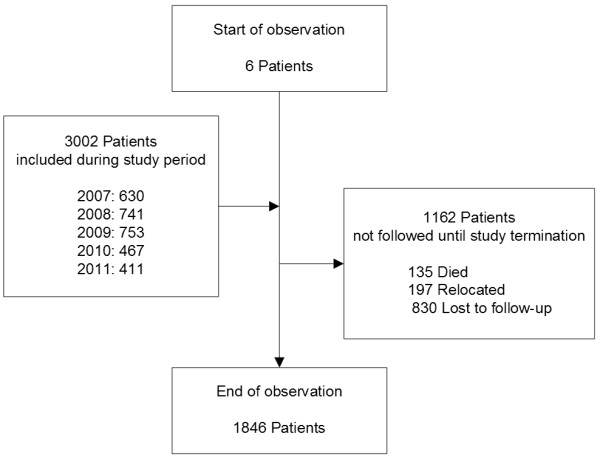
**Flowchart of patient enrollment.** The period of observation lasted from 1st of January 2007 until the 31st of December 2011. All adult (≥ 18 years of age) patients initiating antiretroviral therapy at the study site giving informed consent were included in the analysis. No data on the count of subjects declining informed consent was available.

This analysis included data of 3008 patients (Figure 1). Of these, 2752 (91.5%) had >1 visit recorded, accumulating a total of 5085.2 person-years of follow-up. Median time of follow-up was 1.5 (IQR 0.5-3.0) years. Of these subjects, 1962 (65.2%) were female, median age at enrollment into care was 38 (interquartile range [IQR] 31-45) years and median CD4+ cell count was 168 (IQR 81-273) cells/μL (Table 1). Of the study population included, 1162 patients (38.6%) were not followed until the end of the study in 2011. Of these, 135 (11.6%) died, 197 (16.9%) were transferred to other treatment facilities and 830 (71.4%) were lost to follow-up without indication of the cause (Figure 1). The number of patients initiating cART per year was decreasing after 2009 (Table 2). This decline was temporally related to the opening of alternative treatment facilities in the district during the course of the study.

**Table 1:** Characteristics of study participants at enrollment into care

**Characteristic**		**Value (*n = 3008 *)**
Female sex		1962 (65.2%)
Age (years)		38 (31-45)
BMI (kg/m^2^)		20.3 (18-22.7)
WHO stage at enrollment †	I	949 (31.6%)
	II	776 (25.8%)
	III	808 (26.9%)
	IV	341 (11.3%)
CD4+ count (cells/μl) ‡		168 (81-273)

† missing in 134 (4.4%) ‡ missing in 431 (14.3%).

Data are presented as count (column percentage) or median (interquartile range).

**Table 2:** Proportions of initial first-line regimens prescribed by calendar year

	**2007**	**2008**	**2009**	**2010**	**2011**
	**( *n = 630 *)**	**( *n = 741 *)**	**( *n = 753 *)**	**( *n = 467 *)**	**( *n = 411 *)**
d4T/3TC/NVP	420 (66.7%)	529 (71.4%)	348 (46.2%)	91 (19.5%)	44 (10.7%)
d4T/3TC/EFV	24 (3.8%)	29 (3.9%)	25 (3.3%)	6 (1.3%)	0
AZT/3TC/NVP	8 (1.2%)	10 (1.2%)	5 (0.7%)	20 (4.3%)	57 (13.8%)
AZT/3TC/EFV	178 (28.3%)	173 (23.3%)	320 (42.5%)	154 (33.0%)	137 (33.3%)
TDF/FTC/EFV	0	0	55 (7.3%)	196 (42.0%)	173 (41.9%)

Data is presented as count (column percentage).

### First-line regimen at cART initiation

**Figure 2:**
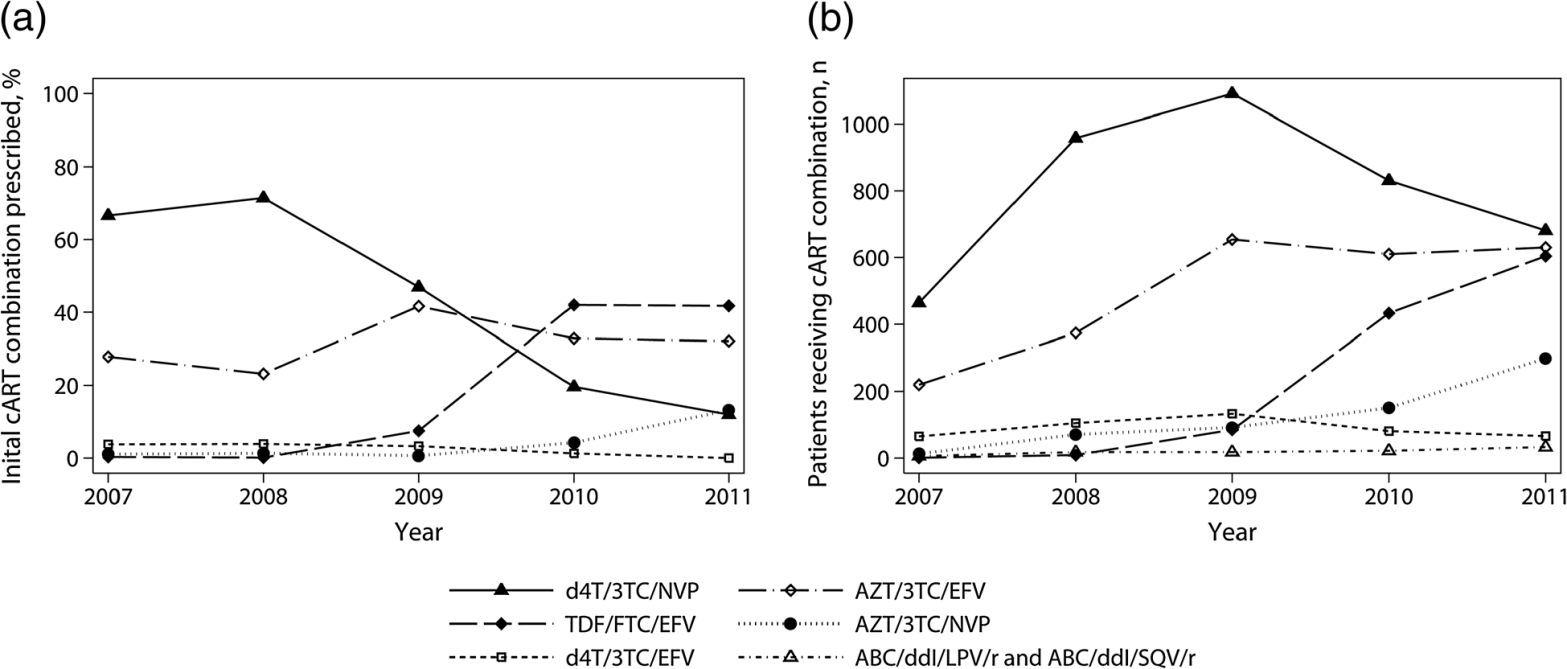
**Line graphs of usage of cART regimens by calendar year. a)** Proportions of initial first-line regimens prescribed by calendar year. **b)** Absolute count of patients being prescribed particular cART drug combinations overall by calendar year.

From 2007 until the end of 2008, d4T/3TC/NVP and AZT/3TC/EFV were the two major combinations used as first-line regimens with proportions of 71.4% and 23.3% in 2008, respectively (Figure 2a, Table 2). A decline of usage of stavudine was observed from 2009 on and only 10.7% of all patients initiating a first-line regimen in 2011 received stavudine (p for trend <0.001). This was compensated initially with increasing usage of TDF/FTC/EFV and AZT/3TC/NVP. The number of initial prescriptions of TDF/FTC/EFV increased rapidly after its introduction in 2009 to 41.9% by the end of 2011. By 2011, the three most commonly used regimens for initiating new patients on cART were TDF/FTC/EFV (41.9%), AZT/3TC/EFV (33.3%) and AZT/3TC/NVP (13.8%).

### Overall cART regimen prescriptions

In every year of observation, d4T/3TC/NVP was the most prescribed combination overall (Figure 2b, Table 3). Still by 2011, 674 (36.5%) patients of this cohort were receiving this regimen but prescriptions were declining from a maximum of 1080 (61.4%) since 2009 (p for trend <0.001). Of those subjects receiving any d4T containing combination in 2011, 659 (89.1%) were initiated in previous years. The second most prescribed regimen was AZT/3TC/EFV, usage of which increased until the end of 2009 to 654 (37.2%) patients and then remained on a constant level. In 2011, 641 (34.7%) of patients were being treated with this combination. Total prescriptions of TDF/FTC/EFV increased from 84 (4.8%) in 2009 to 600 (32.5%) in 2011.

**Table 3:** Absolute count of patients being prescribed particular cART drug combinations overall by calendar year

	**2007**	**2008**	**2009**	**2010**	**2011**
	**( *n = 636 *)**	**( *n = 1274 *)**	**( *n = 1758 *)**	**( *n = 1787 *)**	**( *n = 1846 *)**
d4T/3TC/NVP	456 (71.7%)	955 (74.9%)	1080 (61.4%)	826 (46.2%)	674 (36.5%)
d4T/3TC/EFV	62 (9.7%)	105 (8.2%)	132 (7.5%)	81 (4.5%)	65 (3.5%)
AZT/3TC/NVP	11 (1.7%)	71 (5.5%)	92 (5.2%)	150 (8.4%)	297 (16%)
AZT/3TC/EFV	217 (34.1%)	370 (29%)	654 (37.2%)	609 (34%)	641 (34.7%)
TDF/FTC/EFV	0	0	84 (4.8%)	431 (24.1%)	600 (32.5%)
ABC/ddI/LPV/r / ABC/ddI/SQV/r	6 (0.95%)	18 (1.4%)	18 (1%)	22 (1.2%)	33 (1.7%)

Data is presented as count (column percentage). In case of >1 regimen per year per patient, multiple counts per patients were included in one year. Total number (n) represents sum of patients per year, not sum of regimen counts listed. Column percentages were denominated using *n*.

There were significant differences in the percentage of female and male patients receiving TDF/FTC/EFV in the years 2009-2011. By 2011, only 29.4% of females compared to 36.1% of males received this combination (p = 0.005). On the other hand, the percentage of female patients receiving the nevirapine containing regimens d4T/3TC/NVP and AZT/3TC/NVP was higher than those of male patients but these differences were not statistically significant (in 2011: 38.6% vs. 34%, p = 0.1 and 17.1% vs. 14.9%, p = 0.25, respectively). There was no difference of gender concerning AZT/3TC/EFV (2011: p = 0.82).

The ratio of second-line (i.e. treatment combinations used in case of treatment failure) to total regimens remained stable over time (p for trend = 0.13) with a maximum of 33 (1.7%) patients in 2011. Of these, 31 (93.9%) received a combination of ABC/ddI/LPVr and 2 (6.1%) ABC/ddI/SQVr.

### First-line treatment modifications

Over the whole period of observation, 242 of 1475 (16.4%) subjects receiving any regimen containing stavudine at any point during their follow-up were changed to alternative first-line combinations excluding stavudine after a median of 1.0 (IQR 0.4-1.8) years. The most common replacement was a fixed dose combination of AZT/3TC/NVP which accounted for 40% (97/242) of substitutions overall (Table 4). TDF/FTC/EFV was progressively selected as a replacement for stavudine after 2009 and was the subsequent regimen in 51.1% of all cases in 2011. This rise was mostly compensated by a decline of substitutions to AZT/3TC/EFV, which decreased from 52.2% in 2008 to 7% in 2011. However in 104 cases, stavudine was introduced to the treatment combinations in patients who were not prescribed stavudine in the preceding regimen.

**Table 4:** Subsequent regimens in case a stavudine containing regimen was substituted with another first line combination

	**2007**	**2008**	**2009**	**2010**	**2011**
	***(n = 18)***	***(n = 46)***	***(n = 66)***	***(n = 69)***	***(n = 43)***
AZT/3TC/NVP	2 (11.1%)	22 (47.8)	23 (34.8%)	32 (46.4%)	18 (41.9%)
AZT/3TC/EFV	16 (88.9%)	24 (52.2%)	30 (45.5%)	6 (8.7%)	3 (7%)
TDF/FTC/EFV	0	0	13 (19.7%)	31 (44.9%)	22 (51.1%)

Data is presented in counts (column percentage). In case of >1 regimen per year per patient, multiple counts per patients are included in one year. Total number (n) represents sum of patients per year, not sum of regimen counts listed.

## Discussion

In this rural cohort of HIV-positive patients, modern antiretroviral combinations have been adopted promptly after becoming available and recommended by national guidelines. However, stavudine based combinations were still the most used overall by the end of the year 2011 because 90% of patients who initiated therapy containing stavudine before the update of guidelines in 2009 were not changed to alternative regimens. During the study period, two consecutive editions of the HIV treatment guidelines issued by the NACP of Tanzania were authorative for this cohort. The revision published in 2005 [10] advocated a standard regimen of d4T/3TC/NVP and offered AZT/3TC/NVP, AZT/3TC/EFV as well as d4T/3TC/EFV as alternatives in case of contraindications. In February 2009 [9], the preferred regimen was changed to AZT/3TC/EFV with d4T/3TC/NVP and TDF/FTC/EFV as alternative options. However, changing existing stavudine based prescriptions was only advocated in case of serious side effects. Stavudine was explicitly contraindicated for subjects initiating cART only in the latest revision being issued in 2012 [11], which was after the period analyzed in this study.

As depicted in Figure 2a, the percentage of patients initiated on stavudine containing regimens decreased quickly from a maximum of 71% in 2008 to 11% within the following three years. This scale down conformed to the changed NACP guidelines issued in 2009 and was compensated mostly by increasing prescriptions of AZT/3TC/NVP and TDF/FTC/EFV. Within three years, the new generic fixed dose combination of TDF/FTC/EFV became the most frequently prescribed combination in patients initiating cART with a portion of more than 40% by the end of the study. It is notable that even before the WHO issued explicit recommendations against usage of stavudine in 2010 and several years before the NACP followed suit in 2012, prescriptions of stavudine were decreasing importantly. This occurred supposedly by making alternative regimens available to clinicians observing stavudine-associated adverse events.

In contrast to the changes seen in first-line prescriptions for patients initiating cART, stavudine based combinations were still the most used in the entire cohort by the end of the year 2011. This observation is explained by the fact that patients already receiving stavudine from earlier years on are not changed to more modern combinations in the absence of obvious severe side effects. In this cohort, only 16.4% patient receiving stavudine underwent a substitution to another first-line combination during the period of observation. However, this approach was according to the national guidelines valid for the duration of the study. The WHO recommended exchanging stavudine for alternative regimens from their 2010 guidelines on [1], but acknowledged that this option was limited by cost concerns in low-income settings. Thus far, only few national guidelines in Sub-Saharan African countries, e.g. in Botswana [12], specifically advocated replacing stavudine also in patients not experiencing side effects. Such an approach is sensible and should be sought, as lipodystrophy and peripheral neuropathy develop progressively, even after six years on treatment [2,13]. In 2013, governmental agencies in Tanzania adopted the phasing out of stavudine by completely ceasing the supply of stavudine. A newly available fixed dose combination of tenofovir, lamivudine, and efavirenz is now the preferred recommended first line regimen in Tanzania, and AZT-based combinations have been used as alternative first line regimens.

The WHO published results of their international antiretroviral drug survey in low- and middle-income countries in the HIV report in 2011 [14]. In summary statistics of 14 countries in SSA and India, stavudine based cART constituted the most commonly used one overall, decreasing from a portion of 67% in 2006 to 43% of all regimens in 2010. The decline of the usage of stavudine started two years earlier than in this Tanzanian cohort but prescriptions eventually reached similar levels by 2010. The WHO data showed an increase of prescriptions of TDF-based combinations attaining 15% of total prescriptions by the end of 2010, which is less pronounced than seen in this cohort. However, the WHO presented aggregate data of several countries which does not allow drawing conclusions about trends in individual settings.

TDF/FTC/EFV was less frequently prescribed to women than to men in this cohort. This difference was compensated by a higher rate of prescriptions of d4T/3TC/NVP and AZT/3TC/NVP to female patients. Because of possible teratogenic effects in the first trimester of pregnancy [15], usage of efavirenz was generally not recommended in women of childbearing age [9]. Thus, a lower frequency of regimens containing efavirenz compared to nevirapine as the non-nucleoside reverse transcriptase inhibitor component is plausible. However, it is unclear, why no difference of gender concerning prescriptions of the default regimen AZT/3TC/EFV has been observed.

The rate of patients receiving a second line combination was low (maximum of 1.7% in 2011) when compared to expected therapeutic failure rates [16] which might indicate problems in managing of suspected treatment failure cases. In this cohort, no HIV-1 viral load measurements were available in order to assess eligibility for second line treatment. This lack likely resulted in a less sensitive and specific diagnosis of treatment failure. Proposed prohibitive factors for prescription of second line regimens are distrust against immunological treatment failure criteria and concerns of lack of therapeutic alternatives once second line treatment is initiated [17].

There are several limitations to this study. Due to high rate of patients lost to follow-up, individual follow-up times are short given the overall five year study period, which limits the value of statements about trends in long term therapy. Reasons for treatment modifications were not recorded in a standardized manner. This flaw limits causal analysis of prescription dynamics, particularly those associated with side effects of stavudine.

## Conclusions

Updated guidelines and modern pharmaceuticals have been adopted efficiently in this Tanzanian cohort. Consistent with these, the general usage of stavudine remained very substantial still in 2011: It declined to low levels in antiretroviral combinations of patients initiating cART. However, the majority of subjects being prescribed stavudine primarily in earlier years are not changed to more modern combinations.

Monitoring of drug prescriptions is a simple and feasible approach to identify deficits in implementation of treatment guidelines.

## Abbreviations

3TC: Lamivudine; ABC: Abacavir; AZT: Zidovudine; cART: Combined Antiretroviral Therapy; d4T: Stavudine; ddI: Didanosine; DHHS: US Department of Health and Human Services; EFV: Efavirenz; FTC: Emtricitabine; KIULARCO: Kilombero Ulanga Antiretroviral Cohort; LPVr: Lopinavir / Ritonavir; NACP: National AIDS Control Program; NVP: Nevirapine; SQVr: Saquinavir / Ritonavir; SSA: Sub-Saharan Africa; TDF: Tenofovir; WHO: World Health Organization.

## Competing interests

The authors declare that they have no competing interest.

## Authors’ contributions

FF conceived of the study. FF and EL carried out the analysis of cohort data. FF, EL, TG, CH, MT and MB drafted the manuscript. TG, MT, GM, BJ and CH participated in the design of the study and the KIULARCO cohort. MB supervised the scientific work, participated in design and coordination. All authors read and approved the final manuscript.

## Pre-publication history

The pre-publication history for this paper can be accessed here:

http://www.biomedcentral.com/1471-2334/14/90/prepub
